# Patients Living With Breast Cancer During the Coronavirus Pandemic: The Role of Family Resilience, Coping Flexibility, and Locus of Control on Affective Responses

**DOI:** 10.3389/fpsyg.2020.567230

**Published:** 2021-01-14

**Authors:** Eleonora Brivio, Paolo Guiddi, Ludovica Scotto, Alice V. Giudice, Greta Pettini, Derna Busacchio, Florence Didier, Ketti Mazzocco, Gabriella Pravettoni

**Affiliations:** ^1^Applied Research Unit for Cognitive and Psychological Science, European Institute of Oncology, IRCCS, Milan, Italy; ^2^Department of Oncology and Hemato-Oncology, University of Milan, Milan, Italy

**Keywords:** breast cancer, coronavirus, COVID-19, locus of control, coping flexibility, family resilience, breast cancer patients

## Abstract

The coronavirus disease 2019 (COVID-19) pandemic has strongly affected oncology patients. Many screening and treatment programs have been postponed or canceled, and such patients also experience fear of increased risk of exposure to the virus. In many cases, locus of control, coping flexibility, and perception of a supportive environment, specifically family resilience, can allow for positive emotional outcomes for individuals managing complex health conditions like cancer. This study aims to determine if family resilience, coping flexibility, and locus of control can mitigate the negative affect caused by the pandemic and enhance positive affect in breast cancer patients. One hundred and fifty-four female patients with breast cancer completed the Walsh’s Family Resilience Questionnaire, the Perceived Ability to Cope With Trauma Scale, the Positive-Negative Affect Schedule, and the Mini Locus of Control Scale. Family resilience and internality of locus of control contribute significantly to positive affective responses. Family resilience is responsible for mitigating the negative affect perceived during the pandemic and is enhanced by external locus of control. Evidence suggests that clinical psychologists should develop and propose programs to support oncology patients’ family resilience, coping flexibility, and internal locus of control, allowing for decreased stress and improved adaptability for effectively managing cancer treatment during the pandemic.

## Introduction

Since late February 2020, Italy has been drastically affected by the coronavirus disease 2019 (COVID-19) pandemic, resulting in approximately 229,300 positive cases and 33,000 deaths (World Health Organization, 2020). The pandemic has required a restructuring of the hospital system and suspension of all non-essential health services to better manage the influx of COVID-19 patients while also reducing potential exposure to uninfected patients (Curigliano et al., 2020; van de Haar et al., 2020). The pandemic has also necessitated the implementation of a countrywide lockdown – effective at the beginning of March 2020 – including the closure of places previously open to the public, suspension of all non-essential activities, telecommuting requirements for the workforce, and a stay-at-home order for the general population.

Many COVID-19 patients have presented with comorbidities like cardiovascular disease, liver disease, or malignant tumors (Guan et al., 2020; Thakur, 2020; Yang et al., 2020). However, evidence remains unclear if oncology and other immunosuppressed patients are at an increased risk of severe complications from the virus as compared with healthy individuals among the general population (D’Antiga, 2020; Desai et al., 2020). Oncology patients have been more strongly impacted by the pandemic, not only because of the fear and panic of increased risk of infection (Casanova et al., 2020; Mark and Lewis, 2020; Romeo et al., 2020) but also because many treatment and screening programs have been postponed or canceled until the spread of the virus is stabilized, potentially compromising the affective states of these patients. Donovan stated that when traumatic events like cancer diagnoses or emergency situations arise, personal outcomes – adaptation versus maladaptation – are affected by family response (Donovan, 1998). Additionally, the Double ABC-X model (McCubbin and Patterson, 1983) explains that the interaction between the traumatic event and subsequent life stressors is determined by perceptions of family support, coping strategies, and locus of control.

Family resilience is defined as the perceived ability of a family to withstand a crisis which disrupts their normal course of life and can be a protective factor against stress and negative affect (Walsh, 1996). Perceptions of family’s role, system of beliefs, values, and behaviors are essential in helping a family member overcome traumatic events (Patterson, 2002). For example, if one family member has been diagnosed with cancer, perceived high levels of family resilience can significantly influence personal outcomes such as medication compliance, rehabilitation, and social or occupational reintegration (Faccio et al., 2018). These resources may also offer support in overcoming challenges like negative emotions linked to heightened perception of risk, mandatory quarantine, and possible postponement of treatments presented by the COVID-19 pandemic (Killgore et al., 2020; Prime et al., 2020; Russell et al., 2020).

Another protective factor is coping flexibility. A literature review by Kashdan and Rottenberg (2010) identified coping flexibility as a vital component of health and adjustment to stressors across a variety of settings. Since the early 1990s, coping flexibility has been associated with improved well-being and success in confronting stress (Lester et al., 1994). Additionally, it is associated with decreased anxiety and depression as well as symptom severity, ultimately increasing overall quality of life (Cheng, 2003; Kato, 2012, 2015). These studies demonstrate that coping flexibility contributes to improved psychological well-being, further confirmed by individuals’ reports of decreased levels of depression and increased abilities in managing work-related stress following completion of a coping flexibility intervention (Cheng et al., 2012).

When assuming a situational perspective, a cross-situational view of coping flexibility supports that coping-flexible individuals can adjust their strategies across stressful events (Westman and Shirom, 1995; Murphy, 2001; Thompson et al., 2007). Oncology patients, for example, have already experienced a severe adverse traumatic event – cancer diagnosis and prognosis – which can introduce important emotional consequences (Williams, 2002) and coping challenges (Nipp et al., 2016). Coping flexibility (Oliveri et al., 2019a) is therefore considered a valuable tool that allows such patients to reduce distress (Bonanno et al., 2011) during stressful circumstances (Roussi et al., 2007) like the COVID-19 pandemic. Coping flexibility remains especially vital in reconciling the need to both elaborate the trauma and maintain a positive outlook toward the future after the event has subsided (Bonanno et al., 2008). Oncology patients who can access these coping resources are more likely to endure the pandemic and subsequent lockdown with adequate emotional response (Kaliampos and Roussi, 2018). Examining emotional responses of breast cancer patients during the pandemic can therefore be a preliminary approach to understanding its impact.

Patients’ affect during the COVID-19 pandemic is also influenced by locus of control (Romeo et al., 2019), which determines if individuals perceive the events they are experiencing to be driven by external (e.g., circumstances) or internal (e.g., individuals’ personality, abilities, etc.) factors (Rotter, 1966). These beliefs influence individuals’ cognition, behavior, and affect (Wallston et al., 1978). Evidence has shown that having an internal locus of control is a strong predictor of better psychological adjustment to cancer: behavioral scientists have long been interested in understanding how an individual’s locus of control relates to coping and adjustment (Knappe and Pinquart, 2009; Galvin et al., 2018). Recent research highlights that internal locus of control generates positive emotions and lessens negative ones (Crisson and Keefe, 1988; Gupta et al., 2018). Thus, internal locus of control lowers the perceived level of distress (Ryan and Deci, 2008), threat, and depression (Arraras et al., 2002; Goldzweig et al., 2016) while improving the quality of life (Sharif and Khanekharab, 2017; Toscano et al., 2020) among patients.

The literature therefore highlights the role of these variables in reducing the levels of distress and in promoting psychological adjustment. At the moment, there is no empirical evidence on their impact on Italian cancer patients’ affects during the first month of the 2020 lockdown. Therefore, the purpose of this paper is to determine if family resilience, coping flexibility, and locus of control can mitigate the negative affect and enhance positive affect in Italian oncology patients during the first month of the COVID-19 pandemic.

## Materials and Methods

The study was reviewed and approved by the IEO (Istituto Europeo di Oncologia) Ethic Committee. The patients/participants provided their informed consent to participate in this study.

### Procedure

Participants were eligible for the study if the patient (a) was female, 40–70 years of age at the time of recruitment diagnosis; (b) had a breast cancer diagnosis requiring surgery; (c) is receiving any type of systemic treatment for breast cancer regardless of treatment type; and (d) could both read and speak Italian. All patients received their diagnosis in 2019 or later, and all the patients had cancer in stages I–III. Participants were recruited using a convenience sampling method during the COVID-19 quarantine. Participants were a pool of IEO patients who at the time of the admission gave their availability to participate to research studies and were at the time enrolled in other research projects. All eligible patients were contacted through email or telephone, on the preferred method of contact they gave for the study. A message was included to invite them to take part to the study and an anonymous link to the survey was included.

The survey was hosted by Qualtrics, and an anonymous link was made available to the patients. Participation in the study was voluntary, and patients could withdraw from the study at any time. Participants signed consent forms and completed questions regarding sociodemographic characteristics as well as questions regarding family resilience, coping flexibility in trauma, locus of control, and positive and negative affect. The questionnaire was available from March 19th to March 31st, 2020, starting 4 weeks after the partial lockdown (school closure) and 10 days after the full lockdown (closure of all non-essential businesses and movement restrictions) in Italy.

### Participants

Out of a total of 250 women with breast cancer, 181 started the questionnaire, and 154 completed the questionnaire. Mean age was 51.07 (SD = 7.93). Thirty-seven (20.4%) were single and 146 (79.6%) were in a stable relationship (married or cohabiting), and 127 (70.2%) had children. Regarding educational levels, 40.3% (*n* = 73) of the participants possessed a high school diploma, 7.2% (*n* = 13) achieved a bachelor’s degree, and 26% (*n* = 47) achieved a higher specialization. One hundred and twenty-five participants (69.1%) were still receiving systemic treatment that included chemotherapy, radiotherapy, and/or endocrinological treatment at the time of the survey. All percentages were calculated for total available cases for each demographic variable.

### Measures

After completing the sociodemographic form, participants were asked to fill the following sections:

Family resilience (FR) was measured with the Italian version of the Walsh’s Family Resilience Questionnaire (Rocchi et al., 2017), a 26-item questionnaire, on a five-point Likert scale (1 = “not at all”; 5 = “completely agree”) assessing the three dimensions of family resilience: shared beliefs and support, family organization and interaction, and utilization of social resources. Shared beliefs and support (FR_SBS) includes values, beliefs, and attitudes, establishing a set of shared suppositions that activate emotional responses, form decisions, and orientate action (15 items). Family organization and interaction (FR_FOI) is the perceived family capacity to adapt and identify collaborative solutions to manage crises and avoid conflicts (eight items). Utilization of social resources (FR_USR) indicates individuals’ perceived ability of the family to harness support from social and institutional organizations (three items).

Coping flexibility (CF) was measured with the Italian version of the Perceived Ability to Cope With Trauma Scale (PACT) (Saita et al., 2017), which examines the broad categories of coping behaviors in response to potentially traumatic experiences. The Italian version of the PACT differs from the original one for the number of items and dimensions: The questionnaire was composed of 14 items which asked participants to rate their ability to use different coping strategies on a seven-point scale (1 = “not at all able”; 7 = “extremely able”). Forward focus (CF_FF, nine items) is the component that assesses coping abilities related to maintaining plans and goals, attending to the needs of others, thinking optimistically, remaining calm, reducing painful emotions, and laughing. The trauma focus subscale (CF_TF, five items) explores the ability to fully experience the emotional and cognitive significance of a stressful, and potentially traumatic, event.

Locus of control (LOC) was measured with the Italian version of the six-item Mini Locus of Control Scale (MLCS) (Perussia and Viano, 2008). It is a self-reported scale that investigates locus of control based on three factors: chance (LOC_C, two items), powerful of others (LOC_PO, two items), and internality (LOC_I, two items). The questionnaire is composed of six items on a four-point Likert scale (1 = “not at all”; 4 = “completely agree”).

Affect was measured through the Positive-Negative Affect Schedule (PANAS) (Terraciano et al., 2003). This scale is comprised of two 10-item mood scales and was developed to provide brief measures of positive and negative affect. Subjects are asked to rate each peculiar emotion experienced within a specified time period, with reference to a five-point scale (1 = “very slightly or not at all”; 5 = “very much”). The specified time period in this case was 15 days before the questionnaire was completed, taking place during the COVID-19 pandemic in Italy.

### Statistical Strategy

Demographic variables were described using descriptive statistics. Before proceeding with the regression analyses, assumptions were checked and the data met the requirement for the analysis. Two stepwise regression analyses (forward method with removal test for the least useful predictor) were conducted with dimensions of family resilience, dimensions of locus of control (chance, powerful others, internality), and coping flexibility as predictors, with positive and negative affect as outcome variables. Variance inflation factors (VIF) revealed acceptable values of collinearity between independent variables and are included in Table 2.

## Results

Table 1 shows the mean, standard deviations, and correlations among variables for both the regression outcome variables. Positive affect correlates adequately with all the other variables included in the analysis, except for the chance dimension of the locus of control (LOC_C). Negative affect is significantly correlated with all the variables considered. The chance dimension of locus of control (LOC_C) is not connected with the trauma focus factor of coping flexibility (CF_TF). Other non-correlated variables are two dimensions of locus of control: internality (LOC_I) and chance (LOC_C). Please see Table 1 for detailed results of the correlation analyses.

**TABLE 1:** Means, standard deviations, and correlations for all the variables considered.

	Mean	Std. deviation	1.	2.	3.	4.	5.	6.	7.	8.
Positive affect	2.979	0.778	0.385**	–0.106	−0.178*	0.560**	0.481**	0.499**	0.603**	0.408**
1. Locus of control_internality (LOC_I)	3.302	0.410		0.053	−0.167*	0.402**	0.302**	0.256*	0.276**	0.139*
2. Locus of control_chance (LOC_C)	2.656	0.678			0.375**	−0.155*	–0.085	–0.122	−0.158*	–0.086
3. Locus of control_powerful of others (LOC_PO)	1.753	0.622				−0.280**	–0.095	−0.384**	−0.289**	−0.190*
4. Coping flexibility_forward focus (CF_FF)	5.049	0.920					0.631**	0.490**	0.654**	0.380**
5. Coping flexibility_trauma focus (CF_TF)	5.003	0.871						0.332**	0.438**	0.340**
6. Family resilience_shared beliefs and support (FR_SBS)	56.630	9.345							0.742**	0.532**
7. Family resilience_family organization and interaction (FR_FOI)	28.721	4.758								0.516**
8. Family resilience_utilization of social resources (FR_USR)	9.461	1.961								
Negative affect	2.150	0.710	−0.170*	0.297**	0.413**	−0.399**	−0.138*	−0.419**	−0.481**	−0.241*
1. Locus of control_internality (LOC_I)	3.301	0.409		0.053	−0.167*	0.402**	0.302**	0.256*	0.276**	0.139*
2. Locus of control_chance (LOC_C)	2.655	0.677			0.375**	−0.155*	–0.085	–0.122	−0.158*	–0.086
3. Locus of control_powerful of others (LOC_PO)	1.753	0.621			1.000	−0.280**	–0.095	−0.384**	−0.289**	−0.190*
4. Coping flexibility_forward focus (CF_FF)	5.049	0.919					0.631**	0.490**	0.654**	0.380**
5. Coping flexibility_trauma focus (CF_TF)	5.003	0.870						0.332**	0.438**	0.340**
6. Family resilience_shared beliefs and support (FR_SBS)	56.629	9.345							0.742**	0.532**
7. Family resilience_family organization and interaction (FR_FOI)	28.720	4.757								0.516**
8. Family resilience_utilization of social resources (FR_USR)	9.461	1.960								

N = 154. ** indicates significant values at p < 0.000, * indicates significant values at p < 0.05.

The first stepwise multiple regression was conducted to evaluate whether the dimensions of family resilience, coping flexibility, and locus of control were necessary to predict positive affect. The analyses generated three models (please see Table 2 for details). The final model of the regression analysis accounted for 45.4% of the variance of positive effect with three predictors (family organization and interaction of family resilience, the trauma focus subscale of coping flexibility, and internality of locus of control) with a significant improvement from previous models. Other variables – chance of locus of control (LOC_C: *t* = −0.433, *p* = 0.666), powerful of others of locus of control (LOC_PO: *t* = 0.101, *p* = 0.902), forward focus of coping flexibility (CF_FF: *t* = 1.179, *p* = 0.240), shared beliefs and support of family resilience (FR_SBS: *t* = 1.007, *p* = 0.316), and utilization of social resources of family resilience (FR_USR: *t* = 1.417, *p* = 0.159) – did not enter the model at any stage (refer to model 3 for values reported).

**TABLE 2:** Regression models, beta values and collinearity statistics for the two regression analyses.

		Unstandardized coefficients		Standardized coefficients			*R*	*R*^2^	Model change				Collinearity statistics
		*B*	Standard error	Beta	*t* test	Sig.			F change	Df1	Df2	Sig. F change	VIF
**Outcome: positive affect**													

1	(Constant)	0.0148	0.308		0.481	0.631							
	Family resilience_family organization and interaction (FR_FOI)	–0.099	0.011	0.603	9.308	0.000	0.603	0.363	86.639	1	152	0.000	1.000
2	(Constant)	–0.501	0.338		–1.481	0.140							
	Family resilience_family organization and interaction (FR_FOI)	0.079	0.011	0.485	7.040	0.000							1.273
	Coping flexibility_trauma focus (CF_TF)	0.240	0.062	0.808	3.909	0.000	0.649	0.442	15.28	1	151	0.00	1.237
3	(Constant)	–1.354	0.435		–3.110	0.002							
	Family resilience_family organization and interaction (FR_FOI)	0.074	0.011	0.450	6.616	0.000							1.273
	Coping flexibility_trauma focus (CF_TF)	0.202	0.061	0.226	3.296	0.001							1.294
	Locus of control_internality (LOC_I)	0.365	0.122	0.192	2.999	0.003	0.674	0.454	8.994	1	150	0.003	1.132

**Outcome: negative affect**													

1	(Constant)	4.212	0.309		13.621	0.000							
	Family resilience_family organization and interaction (FR_FOI	–0.072	0.011	–0.481	–6.756	0.000	0.481	0.231	45.647	1	152	0.000	1.000
2	(Constant)	3.241	0.372		8.721	0.000							
	Family resilience_family organization and interaction (FR_FOI	–0.059	0.011	–0.394	–5.595	0.000							1.091
	Locus of control_powerful of others (LOC_PO)	0.342	0.081	0.300	4.252	0.000	0.560	0.313	18.078	1	151	0.000	1.091
3	(Constant)	2.912	0.404		7.216	0.000							
	Family resilience_family organization and interaction (FR_FOI	–0.058	0.010	–0.386	–5.530	0.000							1.094
	Locus of control_powerful of others (LOC_PO)	0.283	0.085	0.248	3.333	0.000							1.242
	Locus of control_chance (LOC_C)	0.150	0.076	0.143	1.983	0.049	0.575	0.317	3.933	1	150		1.167

*Regression models, beta values and collinearity statistics for the two regression analyses.*

The second stepwise multiple regression was conducted to evaluate whether the same variables were necessary to predict negative affect. Three models were calculated (please see Table 2). The multiple correlation coefficient for the final model was 0.57, indicating that approximately 33.1% of the variance of positive affect could be accounted for by family organization and interaction of family resilience, the powerful of others dimension of locus of control, and internality of locus of control, with a significant improvement from the other models. Internality of locus of control (LOC_I: *t* = −0.454, *p* = 0.650), forward focus in coping flexibility (CF_FF: *t* = −1.090, *p* = 0.278), trauma focus subscale of coping flexibility (CF_TF: *t* = 1.117, *p* = 0.266), shared beliefs and support of family resilience (FR_SBS: *t* = −0.459, *p* = 0.647), and utilization of social resources of family resilience (FR_URS: *t* = 0.307, *p* = 0.759) did not enter the model at any stage (refer to model 3 for values reported).

## Discussion

The data highlights that family resilience, coping flexibility, and locus of control contribute significantly in managing the positive and negative affect in patients with cancer during the COVID-19 pandemic in Italy. Results show that one dimension of each considered construct contributes to positive affect levels. Family organization and interaction (FR_FOI) is the main predictor for positive affect, as it is possible that the pandemic required patients and their family to adapt their previous organizational and interactive patterns to cope with the mandatory and prolonged cohabitation necessitated by the lockdown and to discover new ways of managing the patient’s cancer during the public health crisis, as suggested by colleagues (Killgore et al., 2020; Prime et al., 2020; Russell et al., 2020). It is noteworthy that family organization and interaction (FR_FOI) also mitigates negative affect, as its standardized beta value is negative, which likely occurred because patients who perceive higher levels of family organization and interaction (FR_FOI) manage the requirement of the crisis more efficiently and, thus, experience more positive affect and less negative affect. Furthermore, family organization and interaction (FR_FOI) serves as a protective factor from negative emotions, confirming previous findings indicating that positive and negative emotional states can happen during a crisis simultaneously (Fredrickson et al., 2003; Terraciano et al., 2003; Weber, 2010).

Positive states are also enhanced by the Trauma Focus Scale of PACT, which provides that the perceived ability to focus on processing the trauma focus subscale of coping flexibility (CF_TF) is associated with positive states. The ability to focus on trauma helps personal reorganization (Comer et al., 2014; Sahar and Muzaffar, 2017), demonstrating that this result appears to confirm that the focus on elaborating traumatic events, both individually on the trauma focus subscale of coping flexibility (CF_TF) and as a family about organization and interaction (FR_FOI), helps activate a more positive outlook and think realistically about COVID-19 without using strategies of denial and avoidance or feeling overwhelmed. Experiencing positive emotions in the wake of a traumatic event is particularly important, as it allows individuals to evoke powerful changes in their emotional trajectory (Fredrickson, 1998, 2000). The literature highlights how this type of coping can moderate the impact of heightened trauma exposure (Romero et al., 2015; Juanjuan et al., 2020).

As previously mentioned, family organization and interaction (FR_FOI) is also responsible for mitigating the negative affect generated by the pandemic, which is instead enhanced by two dimensions of external locus of control: chance of locus of control (LOC_C) and powerful of others of locus of control (LOC_PO). When persons believe they have no control over a situation and rely on chance or others to decide how to act, they show higher levels of negative emotions. This potentially results from the perceived lack of agency in the course of their lives and could be related to a “learned helplessness condition.” Literature supports that learned helplessness affects personal resilience and distress management (Mikulincer, 1989; Smallheer et al., 2018). Learned helplessness is a consequence of a perception of scarce personal power over the situation and may result in anxiety, depression, and PTSD (Klein and Seligman, 1976; Akca, 2011; Hammack et al., 2012). This is particularly relevant since coping flexibility does not enter the model with negative affect as an outcome. Additionally, a negative locus of control may affect the ability to access personal coping abilities, which allow persons to activate strategies and tactics (Fresco et al., 2006) to individually deal with the negative event and associated negative affect. The perception of good family support and the family’s ability to autoregulate its resources may compensate for these processes of learned helplessness and aid patients through the crisis.

## Conclusion

These considerations are limited. The models explain 44% of the variance of positive affect and 33% of negative affect produced by the pandemic. There may be other variables that moderate or mediate positive and negative affect, such as health locus of control, dyadic support, relationship closeness, emotional carrying capacity, personality traits, and self-efficacy (Cheng, 2003; Fresco et al., 2006).

Another limitation is that affect is a time-limited outcome. It refers to an affective state that may resolve itself in a short time, and requires constant monitoring of patients to verify if especially negative affective states become more persistent as the COVID-19 crisis continues and transform into more stable psychological conditions such as distress, depression, and anxiety or into optimism and positive outlooks. For example, acute stress disorder (ASD) can occur immediately after the traumatic event and last for less than a month (Weber, 2010). Essentially, a person with ASD can present with stress reactions between 2 days and 4 weeks after experiencing a traumatic event (Smith et al., 1999). One more limitation regarding the affect is that in this study it was not possible to measure the emotional effects of cancer alone (e.g., a baseline before the pandemic). It is not possible to clearly differentiate the emotional effects of the pandemic and of the patient’s cancer, even though the participants were asked to refer to their experience about the pandemic in the previous 15 days. Results are therefore to be taken with caution.

Future studies should also consider the contribution of demographic variables (e.g., parental and relationship status) to the emotional well-being of the patients during critical times, such as the present pandemic.

While this evidence is limited, it can be applied to structuring clinical interventions for both the present and near future as well as for avoiding more serious psychological consequences, as suggested by colleagues from Wuhan (Mei et al., 2020). Clinical psychologists should develop and propose programs to support oncology patients’ adjustment and empowerment (Bryant et al., 1999; Bailo et al., 2019), not only during stressful events but also during follow-ups for further monitoring, as suggested by an emergency psychology approach. Interventions, in particular, should address the “patients’ strategies” (Arnaboldi et al., 2020) to organize and regulate their family organization and interactions; to stay in the moment and think concretely about their choices, behaviors, and emotions during the crisis, and to make them feel more pro-active during the crisis in relation to their cancer and the crisis itself (Stephens et al., 2013; Ramezani and Gholtash, 2015; Oliveri et al., 2019b). These foci of attention could result in patients that are less prone to negative affect and are able to make more effective decisions about their cancer effectively during this pandemic.

## Data Availability Statement

The raw data supporting the conclusions of this article will be made available by the authors, without undue reservation.

## Ethics Statement

The study was reviewed and approved by the IEO (Istituto Europeo di Oncologia) Ethics Committee (ID 2612). The patients/participants provided their informed consent to participate in this study.

## Author Contributions

EB and PG run the analyses. EB, PG, and LS wrote the first draft of the manuscript. EB, PG, LS, AVG, DB, FD, and GPe contributed to the literature search. LS, AVG, DB, FD, and GPe handled the data collection. KM and GPr supervised the research. EB acted as corresponding author. All authors contributed to the ideation and design of the research and to the revisions of the manuscript.

## Conflict of Interest

The authors declare that the research was conducted in the absence of any commercial or financial relationships that could be construed as a potential conflict of interest.
